# mRNA Regulation by RNA Modifications

**DOI:** 10.1146/annurev-biochem-052521-035949

**Published:** 2023-04-05

**Authors:** Wendy V. Gilbert, Sigrid Nachtergaele

**Affiliations:** 1 Department of Molecular Biophysics & Biochemistry, Yale University, New Haven, Connecticut, USA; 2 Department of Molecular, Cellular, and Developmental Biology, Yale University, New Haven, Connecticut, USA

**Keywords:** RNA modifications, RNA metabolism, posttranscriptional regulation, epitranscriptome

## Abstract

Chemical modifications on mRNA represent a critical layer of gene expression regulation. Research in this area has continued to accelerate over the last decade, as more modifications are being characterized with increasing depth and breadth. mRNA modifications have been demonstrated to influence nearly every step from the early phases of transcript synthesis in the nucleus through to their decay in the cytoplasm, but in many cases, the molecular mechanisms involved in these processes remain mysterious. Here, we highlight recent work that has elucidated the roles of mRNA modifications throughout the mRNA life cycle, describe gaps in our understanding and remaining open questions, and offer some forward-looking perspective on future directions in the field.

## 1. INTRODUCTION

**Figure 1:**
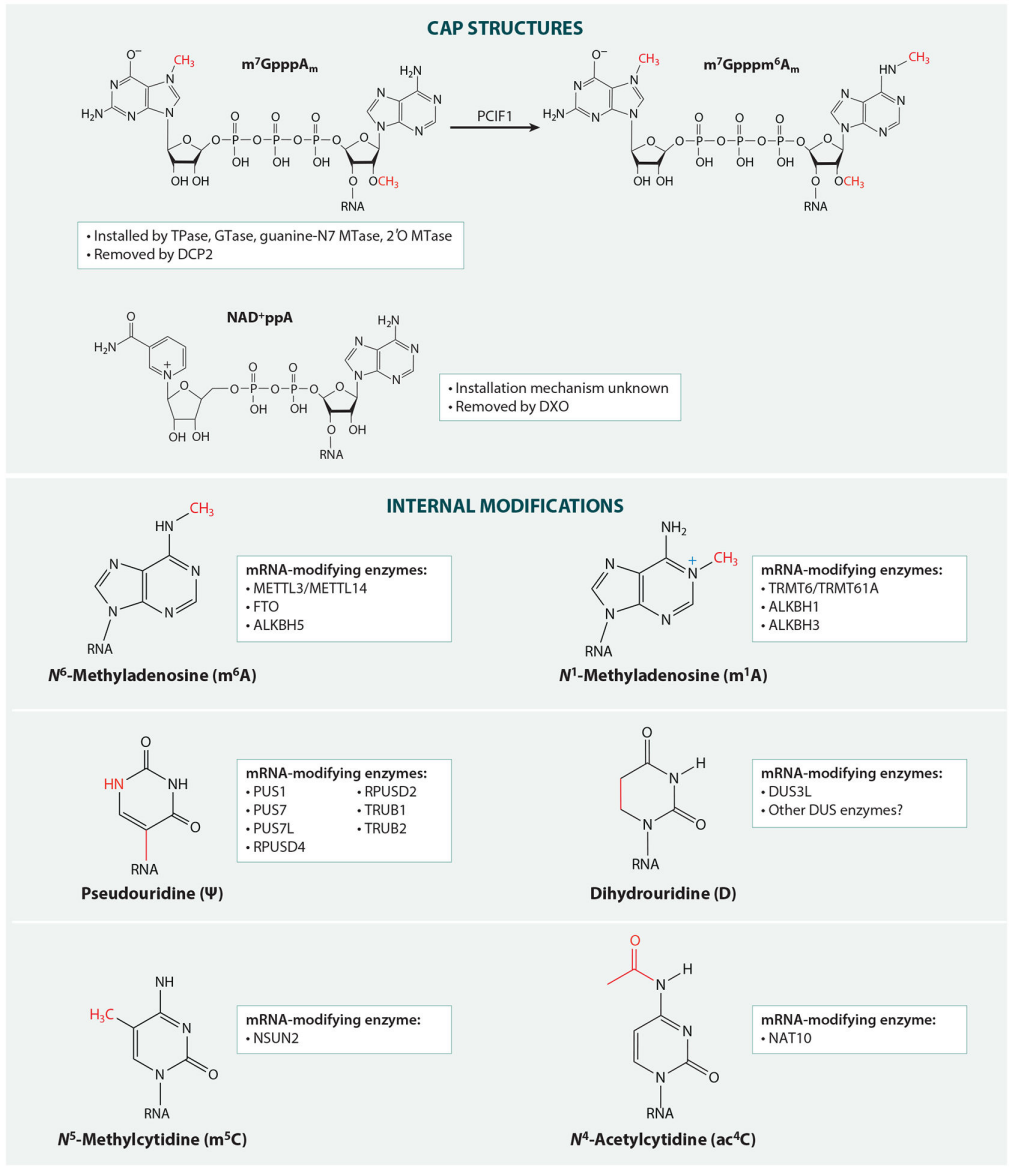
Chemical structures of the modifications described in this review, as well as known enzymes that install and remove these modifications specifically on mRNA. Abbreviations: Ψ, pseudouridine; ac^4^C, *N*^4^-acetylcytidine; ALKBH, alkB homolog; D, dihydrouridine; DCP2, decapping mRNA 2; DUS, dihydrouridine synthase; DXO, decapping exoribonuclease; FTO, alpha-ketoglutarate dependent dioxygenase; GTase, guanylyl transferase; m^1^A, *N*^1^-methyladenosine; m^5^C, *N*^5^-methylcytidine; m^6^A, *N*^6^-methyladenosine; METTL, methyltransferase like; MTase, methyltransferase; NAD^+^ppA, NAD^+^ cap structure with 2′-*O*-methylated adenosine; NAT10, nuclear acetyltransferase 10; NSUN2, NOP2/Sun RNA methyltransferase 2; PCIF1, phosphorylated CTD interacting factor 1; PUS, pseudouridine synthase; RPUS, RNA pseudouridine synthase; TPase, RNA triphosphatase; TRMT, tRNA methyltransferase; TRUB, TruB pseudouridine synthase family.

**Figure 2:**
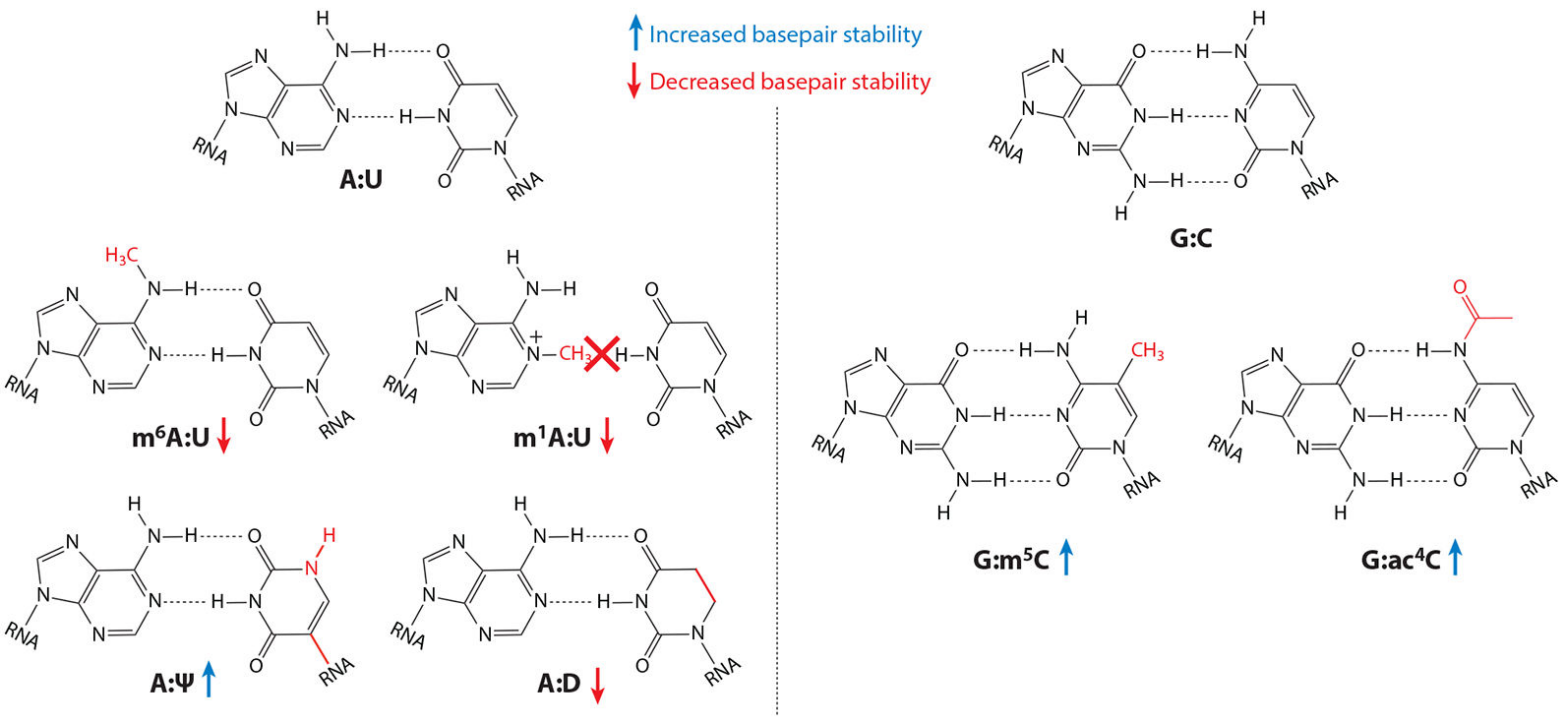
Chemical structures of A:U and G:C base pairs, showing how the chemical modifications m^1^A, m^6^A, Ψ, D, m^5^C, and ac^4^C impact base pairing. Arrows indicate increased (*blue*) or decreased (*red*) base pair stability as a result of the indicated modification. Abbreviations: Ψ, pseudouridine; ac^4^C, *N*^4^-acetylcytidine; D, dihydrouridine; m^1^A, *N*^1^-methyladenosine; m^5^C, *N*^5^-methylcytidine; m^6^A, *N*^6^-methyladenosine.

RNA contains numerous chemical modifications in all organisms. Among the more than 170 chemically distinct RNA modifications known (1), several have been characterized in eukaryotic mRNA. Research on mRNA modifications has expanded exponentially in the past 10 years, fueled by the development of genome-scale methods to map the locations of modified nucleosides such as *N*^6^-methyladenosine (m^6^A), pseudouridine (Ψ), *N*^5^-methylcytidine (m^5^C), *N*^1^-methyladenosine (m^1^A), *N*^4^-acetylcytidine (ac^4^C), dihydrouridine (D), and 2′-*O*-methyl ribose on any nucleoside (Nm) (Figure 1) (for a review of genome-scale approaches to mapping mRNA modifications, see 2-4). Each of these modifications alters the chemical properties of RNA. The chemical changes caused by modifications affect RNA–RNA (Figure 2) and RNA–protein interactions in various ways that are known, or likely, to affect mRNA metabolism. Here, we review recent literature that connects mRNA modifications to effects on steps in the mRNA life cycle from birth to death, including the interplay between transcription and cotranscriptional RNA modification, capping, splicing, cleavage and polyadenylation, export from the nucleus, translation, and decay (for a review of works before 2018, see 5). The extensive literature on m^6^A has been reviewed recently (6, 7) and is covered here only in cases where m^6^A mediates a mechanism of posttranscriptional gene regulation that has not yet been characterized for other mRNA modifications but is likely to occur.

## 2. INTERPLAY BETWEEN TRANSCRIPTION AND RNA MODIFICATION

**Figure 3:**
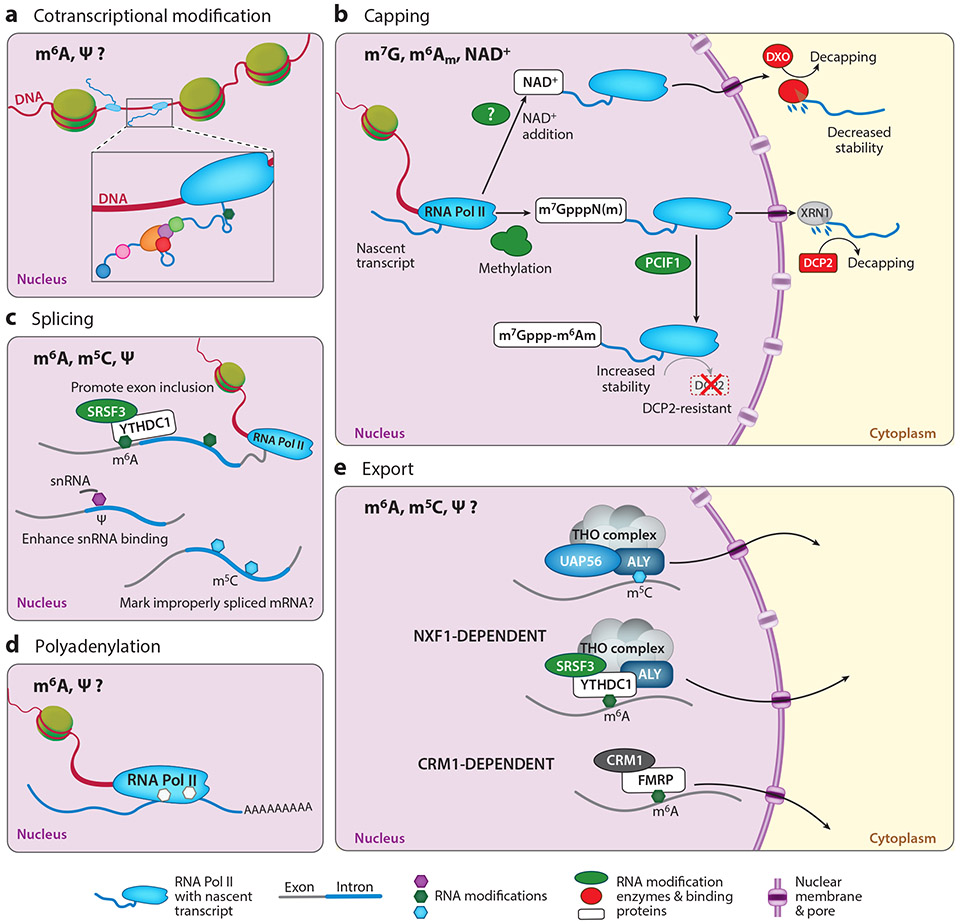
mRNA modifications can be installed cotranscriptionally and regulate multiple steps in nuclear processing and export. (*a*) Some modifications are installed cotranscriptionally via direct recruitment of modification enzymes to RNA Pol II as it synthesizes nascent transcripts. (*b*) mRNA capping involves multiple modifications of the 5′ end of transcripts, which can directly modulate mRNA stability by altering susceptibility to decapping and degradation enzymes. (*c*) Splicing also often occurs cotranscriptionally and has been shown to be regulated by multiple modifications that can alter mRNA–snRNA interactions, recruit proteins that regulate exon inclusion, or mark improperly spliced transcripts. (*d*) Less is known about how mRNA modifications influence polyadenylation, but both m^6^A and Ψ may play important roles. (*e*) RNA modifications likely also influence the nuclear export of properly processed transcripts through both the NXF1- and CRM1-dependent pathways. Abbreviations: Ψ, pseudouridine; ALY, Aly/REF export factor; CRM1, exportin 1; DCP2, decapping mRNA 2; DXO, decapping exoribonuclease; FMRP, fragile X messenger ribonucleoprotein; m^5^C, *N*^5^-methylcytidine; m^6^A, *N*^6^-methyladenosine; m^6^A_m_, *N*^6^-2′-*O*-dimethyladenosine; m^7^G, *N*^7^-methylguanosine; NAD^+^, nicotinamide-adenine dinucleotide; NXF1, nuclear RNA export factor 1; PCIF1, phosphorylated CTD interacting factor 1; RNA Pol II, RNA polymerase II; snRNA, small nuclear RNA; SRSF3, serine and arginine rich splicing factor 3; THO, THO nuclear export complex; UAP56, DExD-box helicase 39B; XRN1, 5′–3′ exoribonuclease 1; YTHDC1, YTH domain containing protein 1.

Key steps in mRNA processing occur cotranscriptionally, including capping, splicing, cleavage, and polyadenylation. Furthermore, proteins that affect later steps in the mRNA life cycle including export from the nucleus, localization in the cytoplasm, translation, and decay are loaded onto nascent pre-mRNAs to assemble functional messenger ribonucleoproteins. It is therefore of interest to know when during mRNA biogenesis RNA modifications are deposited, because this timing constrains the potential effects of RNA modifications on gene expression. Each of the most abundant mRNA modifications is installed by one or more nuclear enzymes, raising the possibility of modification of nascent pre-mRNA at a sufficiently early stage in mRNA biogenesis to affect nuclear processing events and export to the cytoplasm (Figure 3).

### 2.1. Cotranscriptional pre-mRNA Modification

Cotranscriptional deposition has been demonstrated for the two most abundant modifications found in mature mRNA in human cells, m^6^A and Ψ. Ke et al. (8) exploited the stable association of nascent RNA attached to elongating RNA polymerase with chromatin to compare the m^6^A landscape in nascent chromatin-associated pre-mRNA to cytoplasmic mRNA (9). This work concluded that m^6^A is predominantly installed cotranscriptionally, although it did not exclude the possibility of dynamic changes in mature mRNA methylation under different conditions. A different approach used pulse-chase metabolic labeling with bromouridine to show that m^6^A is rapidly installed in nascent pre-mRNA in HEK293 cells (10). The m^6^A methyltransferase complex, METTL3–METTL14, interacts with RNA Pol II (11) and is thus poised to modify nascent pre-mRNA as it emerges from the polymerase. RNA Pol II elongation speed varies across genes (12), and slow elongation through exons may underlie the observed enrichment of m^6^A in exons (13), despite the abundance of potential m^6^A sites (RRACH motifs) in introns (8).

Martinez et al. (14) performed biochemical enrichment of pre-mRNA from the chromatin fraction to show that Ψ is installed cotranscriptionally in human cells. Unlike m^6^A, Ψ is more evenly distributed between exons and introns. Pseudouridine synthase 7 (PUS7) was found to copurify with components of actively transcribed chromatin (15), consistent with its modification of unspliced pre-mRNA. Several additional PUSs were identified as having pre-mRNA targets (14), but the biochemical basis for cotranscriptional recruitment of these enzymes is unknown. It is unclear whether specific recruitment of PUSs to chromatin is necessary to explain the observed distribution of Ψ sites. Notably, all tested PUSs could modify specific intronic target sites in a reconstituted assay containing only minimal RNA and purified PUSs (14). Thus, direct recruitment to elongating RNA polymerase is not strictly required for cotranscriptional RNA modification, although it may increase modification of specific targets, which could contribute to tissue-specific patterns of mRNA modification.

The presence of modified sites within introns provides suggestive evidence that nascent pre-mRNA is a target. Intronic sites were mapped for m^5^C in mouse embryonic stem cells (mESCs) and brain tissue (16), hm^5^C in *Drosophila* S2 cells (17) and mESCs (18), m^1^A in HEK293T cells (19), and D in budding yeast (20). However, most intronic modified sites were mapped in poly(A)-selected RNA, which may include intron-retained mature mRNA. The observation that short-lived RNAs transcribed from enhancers contain m^5^C provides further evidence that cotranscriptional deposition of this modification is likely (21). The TRMT6–TRMT61A methyltransferase that installs m^1^A in mRNA and tRNA is nuclear in human cells and interacts with splicing factors in high-throughput studies (22), but it is not known whether this interaction takes place in the context of nascent pre-mRNA. D was identified in a few introns in poly(A)-selected RNA from *Saccharomyces cerevisiae* (20), but it was not determined when in the mRNA life cycle these intronic sites were modified. For the intron-modified RPL30 gene, deletion of D synthases resulted in a modest accumulation of unspliced RNA, which could reflect impaired splicing or greater stability of unspliced RNA. Application of appropriate mapping techniques to purified chromatin-associated or metabolically labeled nascent RNA will clarify whether intronic m^5^C, hm^5^C, m^1^A, or D modifications are installed cotranscriptionally and whether they have the potential to affect nuclear pre-mRNA processing.

ac^4^C is installed in mRNA by the nuclear acetyltransferase NAT10 (23). Mapping of ac^4^C has been limited to mature poly(A)-selected RNA from total RNA thus far, and ac^4^C has not been reported in introns, which were depleted from the sequenced sample, as expected for mature mRNA. The basis for recruitment of NAT10 to specific targets, nascent or otherwise, remains to be determined. It is plausible that ac^4^C could be introduced in pre-mRNA during transcription, as NAT10 also modifies nascent pre-rRNA (24).

Several factors install 2′-*O*-methyl ribose in noncoding RNA, including the protein-only enzymes FTSJ3, TARBP1/TRMT3, FTSJ1/TRMT7, TRMT13, and TRMT44 and fibrillarin, which is guided to specific targets by base-pairing with C/D-box small nucleolar RNAs (25). The presence of 2′-*O*-methylated nucleosides in poly(A)-selected mRNA is supported by mass spectrometry analysis of human HeLa cells (26) and mouse liver (27). Initial maps have been reported using single-nucleotide-resolution methods (26), but the enzymes responsible have not been determined for the vast majority of mRNA sites. Both fibrillarin and FTSJ3 modify nascent pre-rRNA, consistent with the possibility for cotranscriptional mRNA methylation on ribose. Overall, it is likely that many mRNA modifications in addition to m^6^A and Ψ are deposited co-transcriptionally, endowing them with the potential to influence early steps in mRNA biogenesis. However, determining how this deposition is regulated requires detailed further analysis, as different modification enzymes may interact with the transcription machinery and nascent transcripts via different mechanisms.

### 2.2. Feedback from RNA Modification to Transcription

Transcription and RNA modification are coupled processes when modifying enzymes target nascent pre-mRNA. Functional coupling may occur in the opposite direction via feedback from pre-mRNA modification to the transcriptional machinery. For example, the METTL3–METTL14–WTAP m^6^A methyltransferase complex localizes to promoter and enhancer regions of actively transcribed DNA in human and *Drosophila* cells, and depletion of methyltransferase components or nuclear m^6^A-binding proteins affects transcription (28-30). Notably, tethering METTL3 was sufficient to affect RNA Pol II pausing in *Drosophila* cells, and this was not observed with catalytically dead METTL3, consistent with a direct role for m^6^A in RNA (29).

Mechanistically, m^6^A has been proposed to affect transcriptional regulation by several mechanisms that may generalize to other modifications. Work by Xu et al. (28) uncovered an antagonistic relationship between nascent m^6^A modification and premature transcription termination by the Integrator complex. They proposed that m^6^A deposited near the 5′ end of nascent pre-mRNAs promotes productive transcription elongation by recruiting RNA-binding proteins (RBPs) such as hnRNPG and YTHDC1 that compete with binding by Integrator. m^1^A also binds YTH proteins (31, 32) and so potentially regulates transcription processivity versus premature termination by the same mechanism as m^6^A. In some cells, m^1^A was found to be enriched near the 5′ ends of mRNAs. Other pre-mRNA modifications could suppress or enhance premature transcription termination by affecting binding of various RBPs that compete with Integrator.

RNA modifications may affect transcription through the formation of RNA-dependent phaseseparated condensates at sites of active transcription. Lee et al. (33) linked m^6^A-dependent condensate formation at enhancers to transcription activity. They showed that the nuclear m^6^A-binding protein YTHDC1 formed condensates in vitro that were enlarged in the presence of m^6^A-modified enhancer RNAs (eRNAs) but not unmodified eRNAs of the same sequence. They hypothesized that these condensates could promote the formation of other enhancer-associated condensates, which have been linked to enhancer activity and gene activation (34). Consistent with this hypothesis, the endogenous transcriptional coactivator BRD4 formed reduced numbers of nuclear foci when YTHDC1 or METTL3 was depleted and showed reduced association with enhancer DNA by chromatin immunoprecipitation sequencing. It will be interesting to test whether m^1^A, which also binds YTH proteins (31, 32), is present in eRNAs and similarly promotes condensate formation and gene activation.

The effects of m^6^A on transcription do not always increase mRNA production. m^6^A deposition was associated with stabilization of chromatin-associated regulatory RNAs and proposed to repress transcription by altering the local chromatin state (35). Because nascent chromatin-associated RNAs are short-lived and require specialized sequencing strategies, the landscape of such nuclear RNA modifications is poorly defined. Feedback from nascent RNA modifications to transcriptional output and regulation is a rapidly emerging topic in the m^6^A field that should be explored for other cotranscriptional RNA modifications.

## 3. CAPPING

Processing of the 5′ end of mRNA transcripts is critical for their stability and translation. In eukaryotic mRNA, the majority of transcripts contain a canonical cap containing a terminal *N*^7^-methylguanosine (m^7^G) linked to the next nucleotide via a 5′-to-5′ triphosphate linkage (36). This canonical cap is formed cotranscriptionally via a multistep process that includes an RNA triphosphatase to remove the terminal phosphate, an RNA guanylyltransferase to attach a terminal guanine, and an m^7^G methyltransferase to install the methyl group (37). This canonical cap is often also 2′-*O*-methylated at the first and sometimes second adjacent nucleotide by an m^7^G-specific 2′-*O*-methyltransferase (38). This canonical cap is thought to both stabilize the mRNA 5′ end by protecting it from exonucleolytic cleavage and enhance binding of translation machinery via direct interactions with the eukaryotic initiation factor (eIF) eIF4E (39). Though much less is known about their functions, additional noncanonical cap structures have been identified, including alternative terminal groups such as the metabolite nicotinamide-adenine dinucleotide (NAD^+^) (40, 41), and additional modification of cap-adjacent nucleotides, as in the case of *N*^6^-methylation of A_m_ at the cap +1 position (42).

### 3.1. mRNA Capped with NAD^+^

Though initially identified in prokaryotic RNAs (43, 44), we now know that eukaryotic mRNA can also be capped with NAD^+^ (40, 41). *S. cerevisiae* and human NAD^+^-capped transcripts were identified by taking advantage of NAD^+^ reactivity with ADP ribosylcyclase, which allowed for biotin incorporation via click chemistry and subsequent capture with streptavidin. Both studies demonstrated extensive overlap between the pools of m^7^G- and NAD^+^-capped transcripts, suggesting that there are very few (if any) uniquely NAD_+_-capped mRNAs. Though difficult to quantify, current estimates suggest that approximately 1–5% of a given transcript has an NAD^+^ cap, while the rest likely carries a canonical cap structure (41). The mechanism of NAD^+^ cap addition remains unclear. While it has been demonstrated that both prokaryotic and eukaryotic RNA polymerases can use NAD^+^ to initiate transcription, the fact that small RNAs derived from intron cleavage can also be NAD^+^-capped suggests that a posttranscriptional mechanism also exists (40). NAD^+^ cap removal is better characterized, with the DXO and SpRai1 enzymes identified as the human and *Schizosaccharomyces pombe* NAD^+^-decapping enzymes, respectively (40) (Figure 3). In addition to its decapping activity, DXO has 5′-to-3′ exonuclease activity, which means it can generate its own RNA degradation substrate via its NAD^+^-decapping activity. As a result, removal of these decapping enzymes effectively stabilizes NAD^+^-capped transcripts. While NAD^+^-capped transcripts are not efficiently translated via canonical cap-dependent mechanisms, it remains to be seen whether protein can be synthesized via cap-independent mechanisms. Taken together, NAD^+^-capped transcripts are less stable and inefficiently translated, in direct contrast to the canonical m^7^G cap, which stabilizes transcripts against decay and increases translation efficiency.

Given that the same transcripts can be capped with NAD^+^ or m^7^G, how the distribution of each cap is regulated and how this impacts RNA and protein levels downstream need to be determined. It should be noted that Walters et al. (41) did observe functional enrichment for mitochondrial-encoded transcripts and nuclear-encoded ribosomal protein transcripts and that the levels of NAD^+^-capped mRNAs were higher in yeast grown in synthetic media compared to yeast grown in rich media. Since both mitochondrial function and ribosome biogenesis are sensitive to cellular metabolic state, it is possible that NAD^+^-cap addition serves as an indicator of this metabolic state. Since enzyme-mediated decapping can lead directly to 5′-mediated RNA decay, NAD^+^ could mark transcripts for rapid decay, similar to how uridylation can initiate 3′-mediated decay (45). Taken together, one could envision a regulatory mechanism by which a relative lack of nutrients (e.g., in synthetic media) would trigger increased NAD^+^-capping and subsequent degradation of transcripts related to mitochondrial and ribosomal function, reducing the energy burden on the cell. This is speculative, however, and significant additional work is needed to reveal the mechanisms of NAD^+^-cap installation and function.

### 3.2. Cap-Adjacent *N*^6^-2′-*O*-Dimethyladenosine

In addition to variation at the 5′ terminal position of mRNA transcripts, cap-adjacent nucleotides can also carry additional modifications. The canonical cap is often 2′-*O*-methylated at the adjacent two nucleotides. When the cap-adjacent nucleotide is an A_m_, it can be further *N*^6^-methylated to form m^6^A_m_ (38). This additional methylation renders the cap resistant to DCP2-mediated removal, and initial characterization of this modification suggested that it modulates transcript stability (42). Multiple studies later identified PCIF1 as the methyltransferase that *N*^6^-methylates cap-adjacent A_m_ nucleotides via recruitment to serine-5-phosphorylated RNA Pol II (46-49). Though the identification of PCIF1 facilitated more detailed study of m^6^A_m_ function, these studies came to conflicting conclusions as to whether m^6^A_m_ impacts transcript stability or translation. This may be in part due to confounding variables that need be disentangled: For instance, Boulias et al. (47) showed that while the first transcribed nucleotide of particularly stable transcripts with a half-life >24 h is often m^6^A_m_, the *N*^6^-methylation may not be the cause of but simply correlated with this phenomenon. Further work is needed to identify the correlative versus causative effects and to determine the effect of cap-adjacent m^6^A_m_. But given that it renders transcripts resistant to DCP2-mediated degradation (Figure 3), it is possible that it represents a mechanism to stabilize transcripts beyond the typical mRNA half-life of minutes to a few hours (50).

While these modifications represent just two that diverge from the canonical m^7^G cap, technical advances are revealing additional noncanonical caps in many different organisms (51). It should also be noted that the terminal cap modification, m^7^G, has been identified as an internal mRNA modification by mass spectrometry and sequencing studies (52, 53). While enzymatic tricks were used to isolate internal m^7^G from cap m^7^G, it remains difficult to accurately measure internal m^7^G abundance by mass spectrometry. However, these two studies leveraged both specific chemical reactivity and antibodies to map m^7^G sites and found that the methyltransferase METTL1, in a complex with WDR4, is responsible for internal m^7^G installation. While the prevalence and location of internal m^7^G sites on mRNA remain controversial (54), how such internal m^7^G sites might interact with elongating ribosomes and mRNA-binding proteins remains intriguing.

The chemical diversity of cap structures adds additional layers of complexity to transcriptional and posttranscriptional regulation of gene expression and is likely to have a significant impact on cellular transcriptomes and proteomes. However at present, very little is known about how different caps impact the interactions of mRNA transcripts with the translation machinery and other RBPs. Disentangling the effects of alternative caps on stability and/or translation, as well as distinguishing correlation from causation, is critical to progress in our understanding of these mechanisms. To that end, this is an area that could benefit greatly from biochemical and other in vitro experimental approaches to reveal the molecular players and interactions involved.

## 4. SPLICING

Intron removal by pre-mRNA splicing is an essential step in eukaryotic gene expression. Splicing is also highly regulated in human cells to produce alternative mRNA isoforms that encode distinct protein variants or control different levels of protein expression (55-57). RNA modifications that are installed cotranscriptionally on nascent pre-mRNA have the potential to directly influence splicing by affecting RNA–RNA and RNA–protein interactions (13). The role of pre-mRNA modifications in alternative splicing is only beginning to be investigated for modifications other than m^6^A. In Sections 4.1-4.3, we summarize suggestive evidence for modification-sensitive splicing, highlighting important gaps in knowledge and suggesting next steps for the field. For a more extensive treatment of the literature on m^6^A and splicing, see recent reviews (7, 13).

### 4.1. Evidence Linking pre-mRNA Modifications to Splicing and Recommended Approaches

Widespread changes in alternative splicing have been observed following genetic depletion of RNA-modifying enzymes including the m^6^A methyltransferase METTL3 (7); the demethylase FTO (58); and several PUSs, PUS1, PUS7, and RPUSD4 (14). Direct effects of site-specific pre-mRNA modifications on splicing have been shown in a handful of cases for m^6^A (reviewed in 7, 13) and Ψ (14). Recent work combined nascent RNA labeling with m^6^A immunoprecipitation and sequencing to relate the kinetics of splicing to the locations and extent of pre-mRNA methylation and proposed that m^6^A deposition near splice junctions was associated with faster splicing (10). By contrast, enrichment of m^6^A in introns was associated with slow splicing and alternative splicing. It is not clear whether this correlation reflects a widespread and direct effect of m^6^A on splicing kinetics, or if an upstream event, such as RNA polymerase elongation speed, independently affects m^6^A deposition and splicing efficiency.

The extent to which pre-mRNA modifications directly alter the splicing outcome is unclear from most studies. Typical experiments attempt to correlate changes in splicing following genetic manipulation of RNA-modifying enzymes with the locations of modified nucleotides within or adjacent to alternatively spliced regions of transcripts. However, data are lacking for the locations of most modified nucleosides within nascent pre-mRNA introns where many splicing regulatory elements reside.

Nascent pre-mRNA maps of m^6^A have been determined by two approaches: sequencing of chromatin-associated RNA (8) and sequencing of nascent RNA metabolically labeled with bromouridine (10). The map of Ψ in pre-mRNA from HepG2 cells was limited to highly expressed genes by the high sequence coverage required to distinguish Ψ sites from noisy data: Only ~1% of uridine positions were assessed for the presence of Ψ. Thus, no Ψ data were available for most PUS-sensitive alternatively spliced transcripts. Although antibody-based modification profiling such as for m^6^A has the potential to capture sites in unspliced pre-mRNA, the relative abundance of mature mRNA leads to underrepresentation of intronic regions in profiles of total cellular RNA. Future work combining enrichment of nascent pre-mRNA with enrichment of modified RNA could provide comprehensive maps of intronic pre-mRNA modification.

A limitation of genetic depletion studies to study the role of RNA modifications in splicing is that indirect effects may predominate. This is particularly the case when the RNA-modifying enzyme has been depleted for a long time (e.g., by genetic knockout or by constitutively expressed short hairpin RNAs). Only 20–30% of alternatively spliced exons that responded to METTL3 or FTO expression were found to contain m^6^A (8, 58, 59). Some of these splicing changes may be due to intronic m^6^A sites that were not mapped, but it is likely that many reflect indirect effects downstream of m^6^A-dependent changes in gene expression. Similarly, RNA sequencing showed splicing alterations in cells depleted of ac^4^C (NAT10^−/−^ HeLa cells), but acetylated transcripts did not show more splicing changes than similarly expressed unmodified transcripts (23), suggesting a preponderance of indirect effects of NAT10 on splicing.

The RNA-modifying enzymes that install Ψ, m^5^C, m^1^A, and D in mRNA also modify tRNAs, and the absence of tRNA modifications can affect tRNA stability and function (60). Effects on the tRNA pool are likely to cause many indirect changes to splicing by affecting the production of splicing factors. Rapid inactivation using chemical inhibitors of catalytic activity, such as recently reported for METTL3 (61), is a promising approach to identify direct effects on splicing. A generalizable approach—as inhibitors are currently unavailable for most RNA-modifying enzymes—is the use of genetically encoded protein degrons for acute enzyme depletion prior to RNA sequencing (62). Wei et al. (63) used this strategy to distinguish splicing changes that are direct effects of METTL3 acting on pre-mRNA targets from indirect effects. They found that indirect effects predominate in steady state: Remarkably few alternative splicing events that were detected in METTL3 knockout mESCs (8) were recapitulated by acute degradation of METTL3 protein.

### 4.2. Mechanisms of Modification-Sensitive Splicing

Pre-mRNA modifications are likely to affect splicing by stabilizing or destabilizing RNA–RNA and RNA–protein interactions (13). Most modifications affect the stability of RNA base pairing (3) (Figure 2). Splicing requires base pairing between spliceosomal small nuclear RNAs (snRNAs) and intronic sequences, and Ψ modifications of snRNAs in the regions that base pair with pre-mRNA are known to affect splicing (64). It is plausible that Ψ modifications discovered within 5′ splice site and branch site regions (14) could directly affect splice site recognition by U1 and U2 snRNAs, respectively. In addition, pre-mRNA modifications can affect splicing by altering intramolecular RNA structures, as shown for m^6^A (59).

Clear examples of splicing regulation via modification-sensitive RBPs include regulation of splicing in human cells and in *Drosophila* by the m^6^A-binding protein YTHDC1. YTHDC1 was shown to bind methylated exons and promote binding of the splicing factor SRSF3 to promote exon inclusion (65). The *Drosophila* ortholog of YTHDC1, YT521-B, binds to m^6^A-methylated introns flanking a regulated sex-specific exon in *Sex-lethal* and represses inclusion of that exon (66, 67). YTHDC1 may be a general mediator of m^6^A-sensitive splicing given the broad similarity between splicing changes caused by acute depletion of METTL3 and conditional knockout of Ythdc1 in mESCs (35, 63). Binding of YTH proteins to m^1^A in addition to m^6^A (31, 32) raises the possibility that m^1^A could similarly affect splicing via YTHDC1.

Comparing the locations of pre-mRNA modifications to maps of binding sites for regulatory RBPs is a general strategy to identify candidate regulators of modification-sensitive alternative splicing. For example, m^5^C sites were identified that overlapped binding sites for splicing factors SRSF3 and SRSF4, with SRSF3 showing the most overlapping sites (16). The Encyclopedia of DNA Elements (ENCODE) project has generated a large data set of binding sites for more than one hundred RBPs in two human cell lines, HepG2 and K562, using enhanced crosslinking immunoprecipitation and sequencing (eCLIP) (68). Comparing these RBP maps to pre-mRNA Ψ sites mapped in HepG2 identified hundreds of overlaps including in introns flanking alternatively spliced regions (14).

### 4.3. Effects on Splicing or Nuclear Retention and Stability?

Work by Amort et al. (16) on m^5^C suggests another possible role for intronic RNA modifications: marking improperly spliced RNAs. Substantial fractions of m^5^C sites identified in nuclear poly(A)-selected RNA map to introns in mouse embryonic stem cells (44%) and mouse brain (70%). In contrast, comparatively few intronic m^5^C sites (11% and 3.4% of m^5^C sites mapped to introns in mESCs and brain, respectively) were detected in total cellular poly(A)+ RNA, which is mostly cytoplasmic. This disparity suggests that m^5^C-modified transcripts with retained introns are held in the nucleus and degraded without export to the cytoplasm, or alternatively that they are rapidly degraded upon entrance to the cytoplasm. It will be interesting to determine the point during or after the splicing process at which these intronic m^5^C sites are modified and whether the presence of m^5^C affects their fate of retention in the nucleus and/or rapid decay in the cytoplasm.

Taken together, there are examples suggesting that pre-mRNA modifications can directly impact splicing, motivating further work to expand this to additional modifications. However, studies that rely on genetic knockouts of modification enzymes are prone to indirect effects, due to changes in the kinetics of transcription and the effects of modification loss on other RNA populations such as tRNA, which can impact the production of the splicing machinery itself. Moving forward, acute perturbations to enzyme activity combined with sequencing approaches that specifically target mRNA are essential to distinguishing direct from indirect effects of modifications on pre-mRNA splicing.

## 5. POLYADENYLATION

Nuclear mRNA biogenesis ends with the cotranscriptional cleavage of nascent transcripts followed by addition of a nontemplated poly(A) tail. Processing of the 3′ end is an essential step in mRNA biogenesis that is extensively regulated to produce mRNA isoforms that differ in their 3′ untranslated regions (UTRs). Alternative 3′ UTRs affect most posttranscriptional steps in eukaryotic gene expression, including mRNA localization, stability, translation, and regulation by microRNAs (69). As noted in Section 2.1, m^6^A and Ψ have been established as cotranscriptional modifications of pre-mRNA, and indirect evidence makes it likely that additional mRNA modifications are installed in nascent pre-mRNA. The potential for cotranscriptional pre-mRNA pseudouridylation to affect alternative 3′-end processing was suggested by widespread changes in 3′ UTR isoforms following depletion of PUSs that modify nascent pre-mRNA (14). Although this work did not establish a direct role of specific Ψ in affecting cleavage and polyadenylation, Ψ was identified in the binding sites of cleavage and polyadenylation factors, suggesting a likely mechanism. Analysis of steady-state mRNA isoform abundance as a proxy for alternative cleavage and polyadenylation is confounded by the potential for mRNA modifications to affect transcript half-life. This is of particular concern in the case of a modification like m^6^A, which is known to destabilize transcripts. Overall, the role of cotranscriptional RNA modification in nuclear pre-mRNA processing is understudied.

## 6. EXPORT

Export of mRNAs from their nuclear transcription sites out to the cytoplasm for translation is intricately regulated and requires that many of the preceding processing steps discussed here have been properly carried out. Methylation of both cytidine (m^5^C) and adenosine (m^6^A) have thus far been implicated in the regulation of mRNA export via the NXF1-dependent export pathway (70, 71) (Figure 3). This pathway involves direct mRNA binding by the TREX complex via ALYREF, which functions as an adapter protein. ALYREF itself is a reported m^5^C-binding protein that facilitates the export of m^5^C-methylated mRNAs (71). While ALYREF shuttles between the nucleus and cytoplasm, loss of function of the m^5^C methyltransferase NSUN2 results in retention of more ALYREF in nuclear speckles, which led to the suggestion that loss of m^5^C-binding sites is sufficient to reduce its shuttling to the cytoplasm. While rescue experiments using both wild type and catalytically inactive NSUN2 support this claim, they do not take into consideration the non-mRNA-targeted effects of NSUN2. Nuclear speckles can accumulate RNA-processing factors under conditions that induce cellular stress. Such conditions could include loss of tRNA methylation that results in aberrant tRNA processing or fragmentation, which is likely to occur upon NSUN2 loss (72). In addition, while ALYREF has a higher apparent binding affinity for m^5^C-methylated mRNAs, it does bind unmethylated mRNAs, and mapping studies of ALYREF-binding sites in human cells reveal both motifs that contain C and those that lack C (73). Methylation is also not broadly required for the export of all mRNAs, suggesting that it may alter the export efficiencies of only subsets of transcripts. Therefore, there are likely specific conditions or sequence contexts in which ALYREF regulates the export of m^5^C-containing RNAs that still need to be elucidated.

In addition to m^5^C, m^6^A has also been implicated in the regulation of mRNA export via binding of YTHDC1 (74) and the TREX complex (70). This likely involves a complex containing both YTHDC1 and SRSF3, and knockdown of either YTHDC1 or SRSF3 increases the abundance of m^6^A-containing mRNAs in the nucleus (74). While this initial study did not detect an interaction between either YTHDC1 or SRSF3 and components of the NXF1-export pathway, NXF1 did slightly enrich for m^6^A as measured by immunoprecipitation followed by mass spectrometry of NXF1-bound RNA. Subsequent work, however, suggests that the m^6^A methyltransferase complex recruits the TREX complex to mRNA (70). Simultaneous knockdown of m^6^A methyltransferase complex components KIAA1429 and WTAP results in nuclear retention of m^6^A-methylated transcripts and a reduction of the association of methylated transcripts with the TREX components. In contrast to previous work, this study did detect interactions between YTHDC1, multiple TREX subunits, and NXF1. However, detection of these interactions by immunoprecipitation varied depending on the antibodies used and the configuration of the experiment, suggesting that the antibodies used in the earlier study may have masked the YTHDC1–NXF1 interaction interface. As with m^5^C, m^6^A is not strictly required for mRNA export, and it remains to be determined how and why m^6^A influences the export of some transcripts but not others. Since both the m^6^A- and m^5^C-mediated mechanisms converge on the TREX complex via different adaptor proteins (ALYREF and YTHDC1), it remains possible that other modifications also impact export via a similar mechanism.

Though mRNAs are primarily transported via the NXF1-dependent export pathway, some mRNAs can also be shuttled to the cytoplasm via the CRM1-dependent export pathway that is more typically associated with rRNA and snRNA transport. CRM1-dependent mRNA transport may also be regulated by m^6^A through its interaction with FMRP (75). While the difference in FMRP affinity for m^6^A-methylated versus unmethylated RNA is small, nuclear retention of m^6^A-methylated transcripts can be observed upon *Fmr1* knockout in mice, and *Fmr1* knockout mice phenocopy *Mettl14* conditional knockout mice, suggesting a connection between m^6^A methylation, FMRP, and nuclear export of mRNAs. Work in murine leukemia virus has also demonstrated that both the NXF1 and CRM1 export pathways play important roles in the viral life cycle, and it has been speculated that methylation of viral RNA may play a role in this process as well (76, 77).

While there is tantalizing evidence suggesting that chemical modifications can regulate mRNA export, the reliance on genetic knockout of enzymes that target multiple types of RNA remains a critical issue. Genetic perturbation of enzymes such as NSUN2, which also modify tRNAs, can have wide-ranging impacts on the cellular transcriptome and proteome that can indirectly alter mRNA export. Distinguishing direct interactions between RNA modifications and export machinery from indirect downstream effects is critical for progress in this area. While some tools exist to perturb RNA modifications on specific transcripts (e.g., 78), they vary in efficiency and specificity, so additional technical advances are likely to be required to do this broadly.

## 7. TRANSLATION

The presence of modified nucleosides within mature mRNA invites the question: What do ribosomes do when they encounter modified mRNA? Single modified sites within coding sequences can affect the accuracy and rate of elongation either positively or negatively. This work has been recently reviewed (79). Here we focus on the effects of mRNA modifications on translation initiation, which is highly regulated and usually the rate-limiting step in protein production in eukaryotes.

Translation initiation requires mRNA recognition by eIFs for recruitment of a 48S preinitiation complex (PIC) consisting of a small ribosomal subunit complexed with initiator tRNA and additional eIFs (39). For most mRNAs, recognition of the m^7^G cap promotes ribosome recruitment near the 5′ end of the mRNA, and 48S PICs must scan the 5′ UTR to reach the translation initiation site (TIS). Sequences within 5′ UTRs control the rate of initiation by affecting cap recognition, eIF binding, and scanning of the 48S PIC. Each of the abundant mRNA modifications including m^6^A, Ψ, m^5^C, m^1^A, ac^4^C, and 2′-*O*-methyl ribose has been identified in the 5′ UTR of eukaryotic mRNAs. Modified nucleosides within 5′ UTRs have the potential to affect initiation directly, by affecting interactions with initiation factors and other RBPs, or indirectly, by altering the stability of RNA structures within the 5′ UTR. The effects of m^6^A on translation initiation have been recently reviewed (7). We therefore focus on the known and likely effects of other modifications present within 5′ UTRs, emphasizing ac^4^C as an example for which mechanistic details are emerging.

### 7.1. Position of *N*^4^-Acetylcytidine Within 5′ UTRs Determines Impact on Translation Initiation

**Figure 4:**
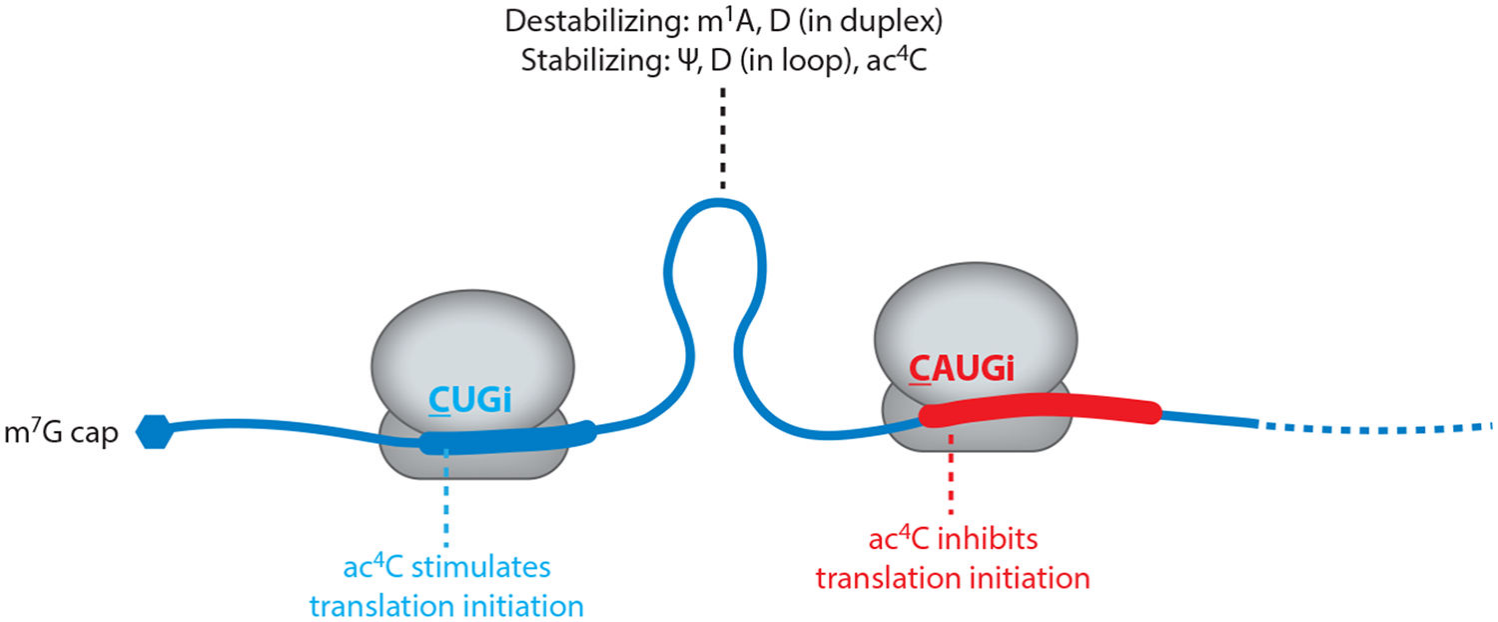
mRNA modifications can regulate translation via multiple mechanisms. Installation of different chemical groups can stabilize or destabilize structured 5′ untranslated regions, which in turn influences the efficiency with which the ribosome can scan those regions. Modifications at canonical (AUG) or near-cognate (CUG) translation initiation sites can also directly impact codon–anticodon interactions. Abbreviations: Ψ, pseudouridine; ac^4^C, *N*^4^-acetylcytidine; D, dihydrouridine; m^1^A, *N*^1^-methyladenosine; m^7^G, *N*^7^-methylguanosine.

Translation assays in vitro and in cells have revealed position-dependent effects of ac^4^C (Figure 4). Initial mapping of ac^4^C using an antibody to enrich modified fragments showed a biased distribution of ac^4^C in HeLa mRNAs with enrichment around translation start codons (23). Subsequent mapping to single-nucleotide resolution identified more than 400 sites in HeLa 5′ UTRs, with estimated occupancy of ac^4^C of ~10–40% based on the RedaC:T-seq signal at rRNA sites known to be acetylated at 80% and 100% from mass spectrometry experiments (80). A substantial fraction (~20%) of the ac^4^C modifications mapped to 5′ UTRs occurred within the ribosome footprint of initiating ribosomes at annotated start sites (80). Nucleotides immediately flanking the initiation codon (AUGi) are known as the Kozak sequence and make specific contacts with initiating ribosomes and eIFs and affect the efficiency and fidelity of start codon recognition (39, 81). Including ac^4^C at −1 and −2 with respect to the AUGi in site-specifically acetylated reporter mRNAs reduced nanoluciferase synthesis by more than 30% in rabbit reticulocyte lysate (RRL) and in transfected HeLa cells. Further experiments showed that endogenous mRNAs with acetylated Kozak sequences, *IRF1* and *KDM4B*, showed increased ribosome density at their initiation codons and increased protein levels in NAT10^−/−^ cells, consistent with a repressive effect of ac^4^C within the Kozak sequence.

The effect of ac^4^C on initiation depended on the specific location of the modified nucleoside. Inclusion of ac^4^C in the 5′ UTR between an upstream TIS and the main TIS decreased protein production, consistent with reduced scanning and increased initiation at a competitive upstream TIS. In contrast, including ac^4^C in a CUG near-cognate initiation codon increased nanoluciferase synthesis from uncapped CUG by more than 50% in RRL and from capped CUG mRNA in transfected HeLa cells. However, ac^4^CUG did not significantly increase nanoluciferase synthesis from capped mRNA in RRL for reasons that were not explained. Although acetylated CUG codons were rare, two ac^4^C sites in upstream TISs with CUG initiation were supported by harringtonine ribosome sequencing of initiating 80S ribosomes (80). It will be interesting to see whether conditions with elevated expression of the mRNA-modifying enzyme NAT10 lead to translational control via increased upstream initiation at acetylated CUG codons within 5′ UTRs.

These reporter studies illustrate the importance of testing site-specific RNA modifications in endogenously modified positions: Moving the ac^4^C by 1 nucleotide—from the C at +1 in a near-cognate CUG initiation codon to −1 upstream of an AUGi—changed the modification from an activator of initiation to an inhibitor. This discovery was made possible by the use of splint ligation to construct site-specifically modified mRNAs for translation in RRL and in HeLa cells by transfecting mRNA. Splint ligation is significantly more demanding technically than incorporating modified nucleosides throughout an mRNA during in vitro transcription, but the results are more informative because physiological mRNA modification is sparse.

### 7.2. Enrichment of *N*^5^-Methylcytidine Near Translation Start Sites

Enrichment near translation start codons is a striking feature of m^5^C maps from diverse organisms and cell types, including in mESCs and brain tissue (16), in primary cultured mouse neurons (82), in CD4^+^ T cells from patients (83), in HeLa cells (84), and in *Arabidopsis thaliana* (85). However, no one has measured the effect of m^5^C in the start codon region on translation initiation with a single, endogenous m^5^C site modified. Delatte et al. (17) showed that full substitution of m^5^C for C reduced production of radiolabeled firefly luciferase protein in RRL by ~50%, which may be due to negative effects of m^5^C on elongation (86). Therapeutic mRNA studies have tested m^5^C-substituted mRNAs and observed increased protein production in some cases, likely due to reduced recognition by innate immune sensors (87). Although m^5^C likely affects multiple steps, this suggests that m^5^C is compatible with reasonably efficient translation initiation in mammalian cells. Because m^5^C is installed in mRNA by enzymes that also modify tRNA, global analysis of the effects of genetically depleting m^5^C on translation (e.g., by ribosome profiling analysis comparing m^5^C-modified to unmodified mRNAs) can provide only suggestive evidence that the presence of the modification in mRNA affects translation initiation. A promising approach to identify specific m^5^C sites that affect translation initiation is to compare modification levels across polysome gradients that separate cellular mRNAs according to the number of translating ribosomes (84). Most sites that showed differential methylation between fractions were less methylated in the well-translated mRNA population, consistent with reduced initiation by mechanism(s) that remain to be explored.

### 7.3. RNA Modifications May Affect Translation by Changing the Stability of 5′ UTR Structures

Modified nucleosides affect the stability of intramolecular RNA folding (Figure 2) (see Section 4.2), which is likely to affect translation initiation. Stable stems within 5′ UTRs impede cap binding, cap-dependent ribosome loading, and scanning to varying degrees depending on their location (Figure 4) (39, 88). Duplex destabilizing modifications such as m^1^A, which blocks Watson–Crick base pairing, could enhance cap accessibility when present near the mRNA 5′ end. By contrast, Ψ stabilizes RNA duplexes by 1–2 kcal/mol (89). The overall effect of a modification on RNA folding depends on the specific location. For example, D disfavors base pairing compared to U but promotes hairpin formation when present in the loop (90, 91).

The relationship between 5′ UTR structure and site-specific RNA modification has been investigated in reporter assays for ac^4^C, which stabilizes RNA duplexes and increases their melting temperature (92). Arango et al. (80) hypothesized that ac^4^C stabilizes an RNA structure that impedes 48S scanning and thereby favors initiation at upstream TISs in weak contexts; consistent with this hypothesis, the inhibition of downstream (main TIS) initiation and nanoluciferase synthesis was observed for a structured 5′ UTR with ac^4^C but not an unstructured 5′ UTR with ac^4^C. Globally, ac^4^C-modified 5′ UTRs (ac^4^C^+^) have more stable predicted folds compared to ac^4^C^−^ 5′ UTRs.

Aided by methodological advances, recent work has begun to reveal what ribosomes do when they encounter modified mRNA. Particularly in the case of translation initiation, the specific position of a given modification can be critical. Further development of approaches to introduce modifications at specific sites is essential to testing the functions of other modifications via in vitro translation experiments. Combining this with cellular experiments comparing modification occupancies across polysome gradients could reveal the involvement of additional modifications in translation initiation.

## 8. STABILITY AND DECAY

mRNA modifications can alter transcript structure and half-life via multiple routes that are not mutually exclusive and can act in concert with one another. The addition of chemical moieties alters RNA chemistry, influencing backbone hydrolysis, base-pairing strength and specificity, and structure (Figure 2) (93). For instance, 2′-*O*-methylation stabilizes the RNA backbone by slowing hydrolysis via 2′ attack on the phosphodiester linkage. In the context of the cell, additional protein factors come into play that influence how chemical modifications alter RNA stability: Modifications can alter susceptibility to nucleases and which binding proteins interact with the RNA in question. m^6^A is an illustrative example of this: m^6^A in the 3′ UTRs of transcripts can recruit binding proteins such as YTHDF2, which in turn can recruit decay machinery to those transcripts (94, 95). This has been reviewed extensively elsewhere (6, 7), so we highlight a few other examples from the recent literature that illustrate these mechanisms.

### 8.1. Stabilization by *N*^5^-Methylcytidine

Early bisulfite-based sequencing studies of m^5^C yielded highly divergent information with respect to its prevalence and distribution (16, 96-98). Methods have since improved and allowed for more mechanistic dissection of this modification (99), and recent studies of m^5^C function now suggest that it can stabilize mRNA. The maternal-to-zygotic transition (MZT) is a critical developmental stage during which expression of maternal mRNAs broadly declines and zygotic genes must be properly activated (100). In zebrafish, m^5^C is installed by the NSUN2 methyltransferase and stabilizes a subset of maternal mRNAs related to mRNA metabolism and cell cycle regulation (101). This effect is due at least in part to the binding of YBX1 to m^5^C-containing mRNAs, which in turn recruits PABPC1 and stabilizes these transcripts. Loss of YBX1 in zebrafish results in developmental arrest at 6 h postfertilization and subsequent lethality, suggesting that m^5^C-mediated maternal mRNA stabilization is critical for proper development. However, YBX1 does have other documented critical roles in development that may be unrelated to m^5^C (e.g., 102). Stabilization of mRNAs by m^5^C has also been observed in urothelial carcinoma of the bladder (UCB). In a recent study of a UCB patient cohort, significantly more m^5^C sites were found in tumor samples relative to controls (103). This higher prevalence of m^5^C correlated with the increased stability of several oncogenes known to drive metastasis and invasiveness. While to date this has been shown only in UCB, the same principle could extend to other cancers as well.

**Figure 5:**
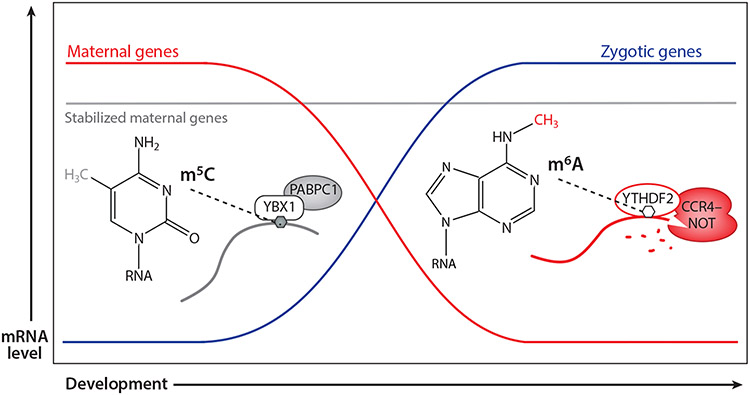
m^6^A and m^5^C can act as opposing regulatory marks during biological processes, such as the maternal-to-zygotic transition. While m^6^A destabilizes maternal mRNAs via recruitment of mRNA decay machinery, m^5^C protects a subset of maternal transcripts from premature degradation by recruiting YBX1 and PABPC1. Abbreviations: m^5^C, *N*^5^-methylcytidine; m^6^A, *N*^6^-methyladenosine.

This m^5^C-mediated stabilization of mRNA transcripts may provide an interesting countermechanism to m^6^A-mediated mRNA decay (Figure 5). Thus far, m^5^C and m^6^A have been characterized in equivalent (or at least similar) biological systems but in separate studies. For instance, m^6^A destabilizes maternal mRNA transcripts during the MZT in zebrafish (104). During the MZT, m^6^A could facilitate the decay of maternal transcripts while m^5^C ensures that a subset of transcripts remain sufficiently stable through the appropriate developmental stages. Similarly, m^6^A has also been shown to destabilize oncogenes in multiple cancers (105, 106), so this interplay between stabilizing and destabilizing modifications could be a more generally applicable mechanism in other biological contexts.

### 8.2. Context-Dependent Effects of Pseudouridine on mRNA Stability?

As described in Sections 4.1, 4.2, and 7.3, recent work has shown that Ψ can affect mRNA splicing and translation. While its potential roles in mRNA stability are still less clear, there remain some intriguing lines of evidence indicating that it may also stabilize mRNA transcripts. Of the initial sequencing studies that mapped Ψ sites in yeast and human transcriptomes (107-109), one study did find that yeast transcripts modified by the Pus7p enzyme had reduced mRNA expression levels in a Pus7p mutant strain (108), suggesting that the presence of Ψ may stabilize at least a subset of transcripts. It has since been demonstrated that human PUS7 regulates alternative splicing [as do other PUS enzymes (14)], so it is possible that changes in the observed equilibrium levels of some Pus7p targets were an indirect result of changes in splicing. Notably, similar Ψ mapping studies in the parasite *Toxoplasma gondii* also revealed a modest but statistically significant change in mRNA stability in strains with the mutated PUS enzyme TgPUS1 (110). However in this case, the effect was in the opposite direction: The presence of Ψ resulted in reduced mRNA stability, and moreover, the effect was not specific to the location of the Ψ site within the transcript (5′ UTR, coding sequence, or 3′ UTR). Thus, while there are some intriguing possible roles for Ψ in the regulation of mRNA stability, more detailed study is required to disentangle the observed effects from its other well-documented functions in mRNA splicing and translation.

While the mechanisms of m^6^A-mediated mRNA decay have now been demonstrated in many contexts, much work remains to reveal the roles of additional modifications in regulating mRNA decay and/or stability. As we have highlighted in other sections, correlation must be distinguished from causality, particularly considering that the stability of a transcript can be influenced by how it was spliced and otherwise processed in the first place. For instance, loss of a modification that influences intron retention would likely also impact the stability of the transcript, without directly interfacing with the decay machinery. A broader investigation of direct interactions between modifications and the decay machinery might yield interesting leads in this regard. The ability to monitor multiple modifications in a single experiment would also be tremendously valuable for directly investigating possible mechanisms of coordination or antagonism within the same biological system.

## 9. CONCLUSIONS AND PERSPECTIVES

Emerging maps of modified nucleosides suggest their potential to affect every step of mRNA bio-genesis, function, and decay. Profiling nascent pre-mRNA has identified m^6^A and Ψ modifications that are installed cotranscriptionally and are poised to influence nuclear RNA processing. Other mRNA modifications are installed by nuclear enzymes, and applying sequencing-based modification profiling to previously uncharted classes of RNA such as pre-mRNA is likely to reveal further examples of modification-sensitive alternative splicing and 3′-end processing. The complete landscape of alternative mRNA 5′ caps is currently unknown and may be complex. New methods and tools are becoming increasingly important for revealing molecular mechanisms, particularly when disentangling direct effects of modifications from indirect and downstream consequences of altering modifications or their regulatory enzymes. In this regard, the development of chemical inhibitors for acute inhibition of additional RNA-modifying enzymes, as recently done for the METTL3 m^6^A methyltransferase, is a promising direction for the field. Detailed mechanistic explanations of regulation by site-specific RNA modifications are few, and more examples are needed to establish paradigms. It is becoming increasingly clear that context matters, as the same modification in different parts of a transcript can have very different functional outcomes. In a similar vein, cellular context also matters. As we dive deeper into mechanisms, more examples of systems where multiple modifications coordinate to regulate cellular processes—such as the opposing effects of m^6^A and m^5^C on mRNA stability—are likely to emerge.

## SUMMARY POINTS

1. Many chemical modifications are likely added cotranscriptionally and can impact all phases of the mRNA life cycle.
2. While many RNA-modification enzymes have been identified, studying their roles via genetic knockout is complicated by the indirect effects of total knockout on cellular transcriptomes and proteomes.
3. Detailed study of specific modifications and transcripts is revealing nuances of RNA-modification-mediated regulation that would otherwise be missed.
4. Continued development of tools to install or remove modifications in a targeted manner is critical to advancing our understanding of the molecular mechanisms of chemical modifications.
